# Supplementary material for: Lamin-Related Congenital Muscular Dystrophy Alters Mechanical Signaling and Skeletal Muscle Growth
Source: Int J Mol Sci. 2020 Dec 30;22(1):306. doi: 10.3390/ijms22010306 (PMC7795708; doi:10.3390/ijms22010306)
Supplement: Supplementary file 1 [file ijms-22-00306-s001.pdf]

Supplementary Figures

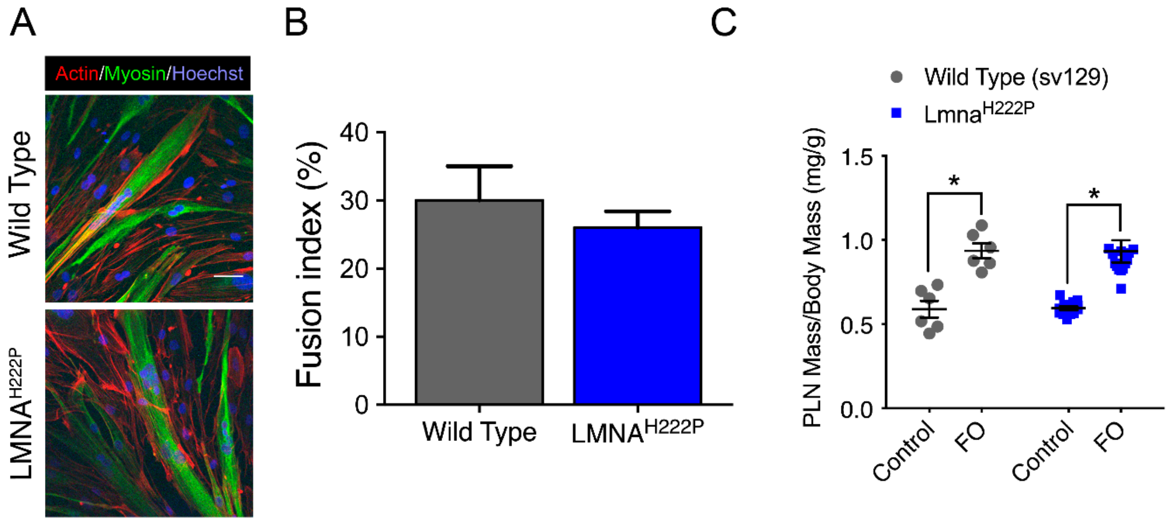

Suppl Fig. 1. Cell culture and plantaris muscle characteristics in Lmna^{H222P} mutation.
(A) Confocal immunofluorescence images of myosin (MF20, green) in WT and *LMNA*^{H222P} cells after 3 days of differentiation. Nuclei are stained with Hoechst (blue). Scale bar=50 μm. (B) Fusion index in WT and *LMNA*-CMD mutant cells after 3 days of differentiation. Values are expressed as means ± SEM. (C) Plantaris muscle mass normalized by body mass from WT and *Lmna*^{H222P} mice in control and after 4-weeks FO. * p<0.05 *versus* control conditions. Values are expressed as means ± SEM.

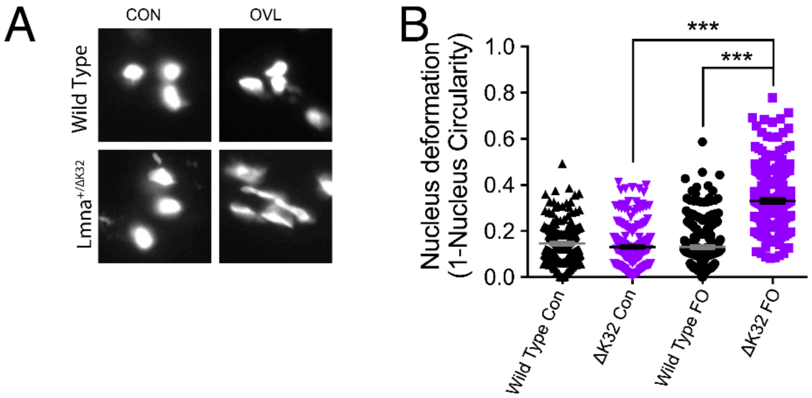

Suppl Fig. 2. Nuclear deformations in Lmna+/ΔK32 mice following functional overload.
(A) Confocal immunofluorescence images of nuclei (Hoechst, white) in WT and *Lmna*+/ΔK32 in control and after 7-days FO. (**B**) Nucleus deformations in WT and Lmna+/ΔK32 in control and after 7-days FO. Values are means ± SEM, n≥230 nuclei/condition.

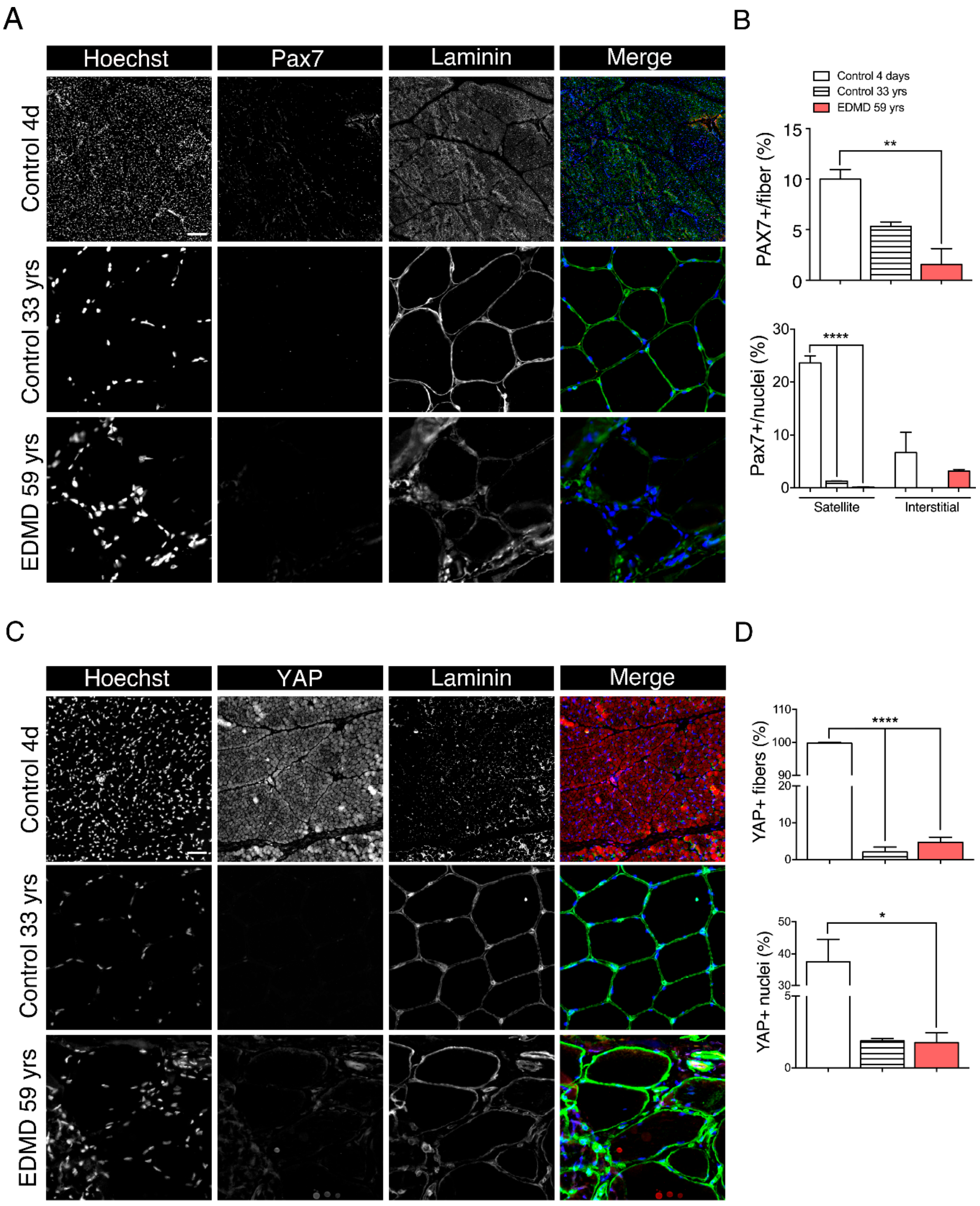

Suppl Fig. 3. Histological data from muscle biopsies of controls and a patient with EDMD.
(**A**) Immunofluorescence images of PAX7+ (red) and laminin (green) in muscle section from a control 4-day-old boy, a control 33-year-old man and an EDMD patient 59-year-old with heterozygous *LMNA*^{H222P} mutation. Nuclei are stained with Hoechst (blue). Scale bar: 30 µm. (**B**) Immunofluorescence images of YAP (red) and laminin (green) in muscle section from section from a control 4-day-old boy, a control 33-year-old man and an EDMD patient 59-year-old with heterozygous *LMNA*^{H222P} mutation. Nuclei are stained with Hoechst (blue). Scale bar: 50 µm. (**C, D**) Quantification YAP+ cells per fibre and YAP+ cells per nucleus in controls and EDMD patient.
